# Investigating the health economic burden of atopic disease in children from the EAT‐On Study

**DOI:** 10.1111/pai.70256

**Published:** 2025-12-04

**Authors:** Ru‐Xin Foong, Joanna Craven, George Du Toit, Helen A. Brough, Alexandra F. Santos, Gideon Lack, Tracey H. Sach

**Affiliations:** ^1^ Department of Women and Children's Health (Pediatric Allergy), School of Life Course Sciences, Faculty of Life Sciences and Medicine King's College London London UK; ^2^ Peter Gorer Department of Immunobiology, School of Immunology and Microbial Sciences King's College London London UK; ^3^ Children's Allergy Service, Evelina London Children's Hospital Guy's and St Thomas' Hospital London UK; ^4^ School of Primary Care, Population Sciences and Medical Education University of Southampton Southampton UK

**Keywords:** atopic disease, food allergy, health economics, healthcare costs, NHS

## Abstract

**Background:**

There is growing interest in the health economic burden of childhood diseases. This study aimed to compare healthcare costs between atopic and non‐atopic children in a general UK population.

**Methods:**

Participants were recruited from the EAT‐On study which followed children originally seen from 3 months old and then 7–12 years. A health economics questionnaire (HEQ) collected data from 2018 to 2022 on the utilization of UK healthcare services and were valued using published unit costs for UK£2021. Mean (standard deviation) resource use and cost were estimated for atopic and non‐atopic children, in addition to mean difference (95% confidence interval) between atopic and non‐atopic children. Two‐part logistic regression analyses were performed to examine the likelihood of a child incurring healthcare costs including variables associated with the level of cost incurred.

**Results:**

625 children completed the HEQ; 33% reported zero healthcare costs over a 12‐month period and 34.4% (215/625) had at least 1 atopic disease. Children with any atopic disease had higher total costs compared to those without atopic disease (mean difference £77 (95% CI −16.9, 170.6) per participant); if extrapolated to population level, this equated to £104.7 million more per year. Children with atopy were more likely to utilize hospital‐based services compared to children without atopy (mean difference £104.3 (95% CI 36.2, 172.5) per participant). Being younger in the 7–12 y age bracket or coming from a lower income household (<£60,000/year) was associated with higher total healthcare costs.

**Conclusion:**

Children with atopy incur greater total healthcare costs compared to children without atopy.

**Abbreviations**
AD: Atopic dermatitis
AIC: Akaike information criterion
EAT Study: Enquiring About Tolerance Study
GP: general practitioner
HEQ: health economics questionnaire
NHS: National Health Service

**Key message**

Children with atopic diseases have higher healthcare costs than non‐atopic children, largely due to greater use of hospital‐based services, indicating an increased health economic impact of childhood atopy in the UK.

## 1 BACKGROUND

There has been increasing interest in understanding the burden of allergic disease for the healthcare sector in terms of cost but also costs borne to patients and their families. The importance of evaluating costs for atopic diseases in children can be related to the prevalence of these diseases in childhood. The prevalence of atopic conditions has been on the rise in the last few decades although varies between populations around the world depending on geographical location and due to the lack of standardization in diagnosing atopic diseases.^1^ For the majority, atopic conditions are managed and well controlled; however, there is still a degree of disease‐related morbidity and disability that exists.^2^ For example, the 2010 Global Burden of Disease survey found that atopic dermatitis (AD) had the highest disability‐adjusted life‐years amongst skin disorders^2^ and allergic respiratory diseases have been recognized as the fourth most important chronic disease according to the World Health Organization.^3^ It is also the case that those with one atopic condition may have another—for example, 10%–40% of patients with allergic rhinitis also have allergic asthma, which can increase the allergic burden of disease.^3^

The understanding of health economics within the context of atopic diseases is still relatively new. Various studies have looked at the cost effectiveness of specific interventions and treatments for asthma,^4^, ^5^ AD^6^, ^7^, ^8^, ^9^ and allergic rhinitis^3^, ^10^, ^11^ or looked at the overall economic burden of food allergies^12^, ^13^, ^14^ but the estimation of the health economic burden of atopic disease in a general pediatric population in the UK has not been done.

The aim of this study was to assess the health economic burden of atopic disease in childhood over a 12‐month period taking a National Health Service (NHS) perspective to further understand the impact atopic diseases have on the UK health system.

## 2 METHODS

### 2.1 Study population

Participants were recruited from the EAT‐On study, which was a follow‐on study of the EAT study. The EAT study was a randomized controlled trial where exclusively breastfed babies were randomized to a standard introduction group (i.e., exclusively breastfed for 6 months before solids were introduced as per standard UK advice) or an early introduction group (i.e., advised from 3 months old to introduce 6 allergenic foods (4 grams of each food protein per week) into their diet).^15^ The EAT‐On study saw any children from the original cohort who were willing to return between ages 7 and 12 years and ran across the time period of 2018–2022. Participants were divided into children who had any atopic disease (i.e., asthma, atopic dermatitis, allergic rhinitis and/or food allergies) and those who did not have atopic diseases. The EAT‐On study was approved by the Health Research Authority London – Chelsea Research Ethics Committee (Ref: 17/LO/1687). Written informed assent and consent were obtained from the participants and their legal guardians.

### 2.2 Health Economic Questionnaire

A Health Economic Questionnaire (HEQ) was developed to collect resource use data from the EAT‐On study population and was modified and adapted from the FA‐ECOQ (see Appendix S1).^16^ The HEQ covered two broad categories—the cost of healthcare services (community services, hospital services such as outpatient, inpatient and emergency services including prescriptions) and the cost of living for children and their families (i.e., expenditure on food, household goods and indirect costs such as time off work/school due to illness) over the last 12 months from the date of HEQ completion. Prescriptions were based on the number of dispensings based on parent recall rather than types of medication unless specifically asked (i.e., adrenaline devices). During the development process, families of allergic patients in our department were consulted and shown the questionnaire for feedback on the content and length of the HEQ with adaptations made based on this feedback. In this paper we focus on the former healthcare service use and costs only. The HEQ was distributed online using Snap 11 WebHost (2010–2020 Snap Surveys Ltd.©) during the child's EAT‐On Study visit between 2018 and 2022, which reduced missing data by forcing an answer for almost all questions to complete the questionnaire. Answers for questions deemed sensitive by the ethics committee (i.e., reporting household income, receiving government benefits) were not forced. All HEQs fully submitted via the SnapSurvey link were included for analysis. Partial or incomplete surveys were not included in this analysis. HEQ was sent either retrospectively if the child had already attended their EAT‐On clinical study visit or before their upcoming visit. Reminder emails were sent out to participants at 2 and 4 weeks after the original date they first received the HEQ if not completed. Follow‐up reminders were given during subsequent face‐to‐face visits. The HEQ was approved for distribution by the ethics committee as Substantial Amendment 4 (Ref: 17/LO/1687).

### 2.3 Statistical Analyses

Data analysis was performed using Stata Statistical Software Release 17 College Station, TX. StataCorp LLC. Participant clinical characteristics and healthcare resource use were compared between atopic versus non‐atopic children descriptively. Mean (sd) healthcare service costs per participant were calculated from various published sources of unit costs, as shown in Table S1, and reported in UK£2021. A total NHS healthcare cost for each participant was estimated with the mean (sd) total NHS healthcare cost presented along with the mean (95% CI) difference between the atopic and non‐atopic groups of children.

A two‐part regression model was used to look at discrete and continuous outcomes with the first part looking at covariates associated with having positive or zero NHS costs and a second part being run to look at covariates that were associated with the scale of positive costs. Covariates that were not significant were removed (backward stepwise approach) and the model was re‐run. The Akaike information criterion (AIC) was used to select the best fitting model.

## 3 RESULTS

### 3.1 Study population

A total of 947 participants were enrolled in the EAT‐On study; 66% (625/947) of these participants completed the HEQ and were included in this study. There were no significant differences between sex, ethnicity or history of atopy between the children who completed the HEQ and those who did not (see Table S2). Of the included participants, 215 (34.4%) reported a history of at least one atopic condition (i.e., AD, food allergies, asthma and/or allergic rhinitis) and 410 (65.6%) did not have any atopic conditions. There were 58 (9.3%) children who reported food allergies and within this group, 42 (72.4%) had AD, 18 (31%) had asthma and 29 (50%) had rhinitis. The proportion of females in the atopic group was significantly higher compared to the non‐atopic group (58.1% vs. 49%, respectively (*x* ^2^ = 4.7, *p* = .03)). There were no other significant differences between the two groups in terms of their clinical characteristics (see Table S3).

### 3.2 Total NHS resource use and costs

**FIGURE 1:**
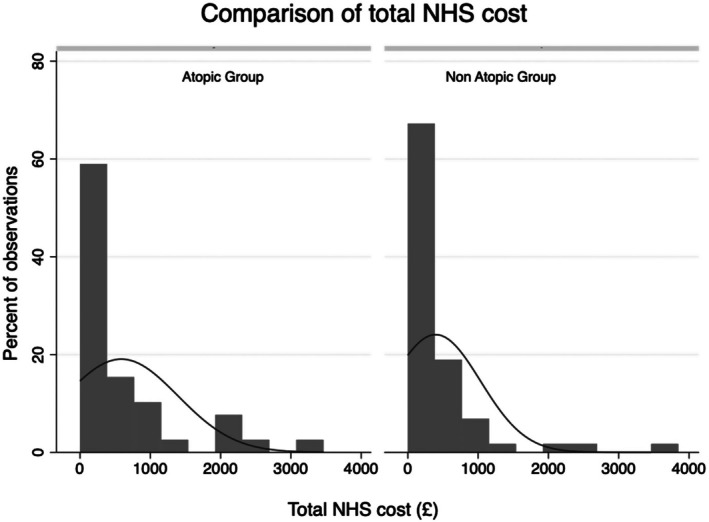
Histogram comparing the total NHS healthcare cost of atopic versus non‐atopic children in this cohort of children.

Total NHS costs including community and hospital services per participant were calculated and showed that atopic children had a higher mean cost compared to those without any atopy, but this was not statistically significant (£316.81 vs. £239.96 (*p* = .11), respectively). For all the patients included in this study, Figure 1 shows a histogram of total cost to the NHS of which the distribution is highly skewed to the right. Of the entire cohort, 36.5% (228/625) had zero total NHS costs. There were 25.6% (55/210) of the children with atopy who had zero NHS costs compared to 42.2% (173/410) of the children without any atopy.

### 3.3 Community services and costs

Children with atopy had a higher mean number of community visits to any healthcare professional compared to those without atopic disease, which was statistically significant (0.62 visits vs. 0.47 visits, *p* < .001). Children with atopy also had a significantly higher number of visits to the general practitioner (GP) and practice nurse in comparison to those without any atopic diseases (1.1 vs. 0.63 *p* < .05 and 0.34 vs. 0.12 *p* < .05, respectively, Table 1). Children with an atopic condition had a higher mean number of prescriptions in the community compared to the non‐atopic children (1.7 vs. 0.5, respectively, *p* < .001, Table 1). There was also a significant difference in the number of community psychology/counseling visits with more children in the non‐atopic group attending these visits compared to those with atopic disease (0.35 visits vs. 0.07 visits, *p* < .05), respectively.

**TABLE 1:** Mean (sd) number of healthcare visits and prescriptions between atopic and non‐atopic children.

Type of healthcare service	Total cohort (*n* = 625)	Any atopy (*n* = 215)	No atopy (*n* = 410)	Mean difference (95% CI)
	Mean (sd)	Mean (sd)	Mean (sd)	
Community services (number of visits)				
Any community healthcare professional (HCP) visit	1.48 (0.5)	1.38 (0.5)	1.53 (0.5)	**−0.15 (−0.23, −0.07)**
GP visit	0.79 (1.2)	1.10 (1.4)	0.63 (1.1)	**0.47 (0.26, 0.69)**
Practice nurse visit	0.20 (0.6)	0.34 (0.8)	0.12 (0.5)	**0.22 (0.11, 0.34)**
Community physiotherapist	0.03 (0.3)	0.05 (0.3)	0.02 (0.3)	0.03 (−0.03, 0.08)
Community occupational therapist	0.02 (0.2)	0.01 (0.2)	0.02 (0.3)	−0.01 (−0.04, 0.03)
Community dietitian	0.00 (0.1)	0.01 (0.1)	0.00 (0.0)	0.01 (−0.04, 0.02)
Community psychology/counseling	0.25 (1.7)	0.07 (0.6)	0.35 (2.1)	**−0.21 (−0.38, −0.04)**
Other community HCP	0.09 (0.3)	0.13 (0.4)	0.08 (0.3)	0.05 (−0.01, 0.12)
Community prescriptions (number of prescriptions)	0.94 (2.2)	1.84 (2.9)	0.48 (1.6)	**1.36 (0.94, 1.78)**
Hospital services (number of visits)				
Any hospital healthcare professional (HCP)	0.20 (0.4)	0.27 (0.4)	0.17 (0.4)	**0.10 (0.03, 0.17)**
Allergy clinic	0.04 (0.3)	0.11 (0.5)	0.00 (0.0)	**0.11 (0.05, 0.17)**
Respiratory clinic	0.02 (0.3)	0.06 (0.4)	0.00 (0.0)	**0.06 (0.00, 0.12)**
Dermatology clinic	0.03 (0.2)	0.06 (0.3)	0.01 (0.1)	**0.05 (0.01, 0.09)**
Gastroenterology clinic	0.03 (0.2)	0.10 (0.3)	0.02 (0.2)	0.04 (−0.00, 0.09)
ENT clinic	0.05 (0.4)	0.04 (0.2)	0.05 (0.4)	−0.01 (−0.06, 0.04)
General pediatric clinic	0.11 (0.4)	0.11 (0.5)	0.10 (0.4)	0.01 (−0.07, 0.09)
Hospital nurse‐led clinic	0.03 (0.3)	0.03 (0.4)	0.02 (0.3)	0.01 (−0.05, 0.06)
Hospital dietitian	0.03 (0.3)	0.06 (0.4)	0.02 (0.2)	0.04 (−0.01, 0.10)
Hospital physiotherapist	0.05 (0.4)	0.08 (0.5)	0.03 (0.3)	0.05 (−0.03, 0.13)
Hospital occupational therapist	0.01 (0.1)	0.00 (0.0)	0.01 (0.2)	−0.01 (−0.03, 0.01)
Hospital psychology service	0.04 (0.4)	0.06 (0.4)	0.03 (0.3)	0.03 (−0.04, 0.10)
Other hospital HCP	0.06 (0.3)	0.07 (0.2)	0.06 (0.2)	0.01 (−0.03, 0.06)
Hospital prescriptions (number of prescriptions)	0.10 (0.6)	0.17 (0.7)	0.06 (0.5)	**0.11 (0.00, 0.22)**
Any Accident and Emergency (A&E) (number of visits)	0.19 (0.5)	0.23 (0.6)	0.17 (0.5)	0.06 (−0.03, 0.15)
A&E prescriptions (number of prescriptions)	0.19 (0.5)	0.21 (0.5)	0.17 (0.5)	0.04 (−0.04, 0.12)
Admissions to hospital				
Inpatient NHS hospital admissions	0.02 (0.2)	0.06 (0.2)	0.01 (0.1)	**0.05 (0.02, 0.08)**
Planned hospital admissions	0.05 (0.4)	0.12 (0.6)	0.01 (0.1)	**0.11 (0.02, 0.19)**
Emergency hospital admissions	0.04 (0.3)	0.09 (0.4)	0.01 (0.2)	**0.08 (0.03, 0.14)**

*Note*: Bold values are statistically significant.

Mean cost per participant was estimated for healthcare services used in the community and hospital but also overall in terms of total National Healthcare Service (NHS) healthcare cost (Table 2). Children with atopy had a higher mean cost in terms of seeing their GP or practice nurse (£43.06 vs. £24.59 (*p* < .001) and £4.82 vs. £1.71 (*p* < .001), respectively). There was also a significant difference in mean cost for community psychology/counseling services in that children without atopic disease had a higher cost compared to those with atopic disease in terms of using this service, £77.93 vs. £15.70 respectively (*p* = .014).

**TABLE 2:** Mean (SD) costs per participant (UK£2021) for different healthcare services.

	Total cohort (*n* = 625)	Any atopy (*n* = 215)	No atopy (*n* = 410)	Mean difference (95% CI)
	Mean (sd)	Mean (sd)	Mean (sd)	
Community services				
GP cost	30.94 (48.4)	43.06 (54.4)	24.59 (43.6)	**18.47 (10.04, 26.90)**
GP nurse cost	2.78 (8.3)	4.82 (10.8)	1.71 (6.3)	**3.11 (1.53, 4.69)**
Community physiotherapist	3.28 (34.0)	5.30 (39.3)	2.22 (30.8)	3.08 (−2.99, 9.15)
Community occupational therapist	2.82 (38.9)	2.23 (24.4)	3.2 (44.6)	−0.89 (−6.31, 4.53)
Community dietitian	0.29 (5.2)	0.86 (8.9)	0.00 (0.0)	0.86 (−0.33, 2.05)
Community psychology/counseling	56.52 (392.8)	15.70 (139.2)	77.93 (473.2)	−62.23 (−111.78, −12.68)
Other Community HCP costs	9.89 (41.6)	8.21 (35.3)	10.76 (44.6)	−2.55 (−8.95, 3.85)
Total cost of community service use	106.01 (407.4)	79.41 (173.6)	£119.96 (486.7)	−40.55 (−93.17, 12.08)
Hospital services				
Allergy clinic	10.75 (79.4)	31.24 (133.2)	0.00 (0)	**31.23 (13.33, 49.14)**
Respiratory clinic	5.49 (39.1)	15.96 (114.4)	0.00 (0)	**15.96 (0.58, 31.35)**
Dermatology clinic	5.36 (51.7)	11.91 (60.5)	1.92 (19.4)	**9.99 (1.64, 18.34)**
Gastroenterology clinic	7.65 (53.6)	14.45 (77.0)	4.08 (35.2)	10.37 (−0.52, 21.26)
ENT clinic	7.38 (55.4)	5.92 (37.2)	8.14 (62.9)	−2.23 (−10.10, 5.64)
General pediatric clinic	32.00 (136.7)	33.82 (142.5)	£31.03 (133.8)	2.78 (−20.31, 25.88)
Hospital nurse‐led clinic	5.00 (60.9)	5.98 (65.0)	4.48 (58.7)	1.50 (−8.91, 11.91)
Hospital dietitian	3.60 (27.9)	6.47 (41.5)	2.09 (16.6)	4.38 (−1.42, 10.19)
Hospital physiotherapist	5.71 (48.7)	9.96 (65.3)	3.48 (37.1)	6.48 (−3.00, 15.96)
Hospital occupational therapist	0.94 (17.0)	0.00 (0.0)	1.44 (21.0)	−1.44 (−3.48, 0.60)
Hospital psychology service	8.88 (82.4)	13.42 (96.2)	6.50 (74.2)	6.93 (−7.86, 21.71)
Other Hospital HCP costs	11.89 (56.2)	14.95 (63.8)	10.29 (51.7)	4.66 (−5.27, 14.59)
Accidental and Emergency (A&E) visits	57.02 (149)	69.07 (170.3)	50.71 (136.2)	18.36 (−8.03, 44.76)
Planned admissions to hospital	243.35 (1965.6)	609.83 (3208.5)	51.17 (633.2)	**558.67 (123.07, 994.27)**
Emergency admissions to hospital	144.07 (956.4)	335.05 (1438.6)	43.92 (532.5)	**291.12 (91.05, 491.19)**
Total cost of any hospital admissions	387.42 (2670.2)	944.88 (4237.4)	95.09 (1110.9)	**849.79 (270.21, 1429.37)**
Total cost of any hospital service use	149.77 (358.8)	218.21 (464.8)	113.88 (282.1)	**104.33 (36.19, 172.47)**
Total cost of prescriptions				
Any community prescriptions	8.17 (19.3)	15.89 (25.4)	4.11 (13.6)	**11.78 (8.12, 15.44)**
Community – prescriptions for AAI	6.12 (29.9)	17.79 (49.0)	0.00	**17.79 (11.1, 24.4)**
Any hospital prescriptions	0.83 (5.0)	1.44 (6.1)	0.51 (4.2)	**0.94 (0.02, 1.86)**
Hospital – prescriptions for AAI	1.83 (14.8)	5.31 (25.0)	0.00	**5.31 (1.9, 8.6)**
Any A&E prescriptions	1.62 (4.5)	1.85 (4.3)	1.50 (4.6)	0.35 (−0.37, 1.08)
Total Mean cost per participant for prescriptions (£)	10.62 (22.6)	19.19 (28.7)	6.12 (16.9)	13.07 (8.88, 17.26)
Total NHS cost (Any healthcare service use: community, hospital, prescriptions)	266.40 (583.0)	316.81 (549.3)	239.96 (598.8)	76.85 (−163.9, 170.64)

*Note*: Bold values are statistically significant.

### 3.4 Hospital services and costs

Children with atopy had a significantly higher number of mean visits to any type of hospital healthcare professional compared to children without atopy (0.3 vs. 0.2 visits, respectively, *p* < .01), especially in terms of attendance at specialty hospital clinics including pediatric allergy, respiratory and dermatology clinics (*p* < .05) (Table 1). Atopic children were significantly more likely to have inpatient hospital admissions, whether it was a planned or an emergency admission (0.06 admissions vs. 0.01 admissions, *p* < .05). There were no significant differences in attendance at A&E between the two groups.

There was a significant difference between the two groups when it came to the total cost of any hospital service use (including outpatient clinics, A&E visits, hospital admissions) with the mean cost of services being higher in the children with atopic disease than those without (£218.21 vs. £113.88, *p* = .003). Atopic children were significantly more likely to attend specialist hospital clinics such as allergy (*p* = .001), respiratory (*p* = .042) and dermatology (*p* = .019). There were no significant differences between the mean cost of attending A&E. Children with atopic disease also had a greater mean prescription cost compared to the non‐atopic children (£19.19 vs. £6.12 (*p* < .001), respectively) and this was still relevant if the prescriptions were broken down to community or hospital prescriptions.

The two‐part regression analysis identified covariates associated with whether an individual had positive or zero NHS costs (Table 3). Being younger and having any atopic diseases were associated with a higher probability of having positive NHS costs. In the second part of the analyses, the higher the annual income bracket, the lower the total NHS cost with those in the £36,001–60,000 salary bracket incurring significantly lower total NHS costs (−£196.5, 95% CI −376.9, −16.1) and those earning more than £60,000 per annum incurring a lower total NHS cost of −£253.8 (95% CI −433.9, −73.8) compared to the lowest income bracket.

**TABLE 3:** Backwards stepwise regression model looking at variables associated with total NHS healthcare cost.

Independent variable	Coefficient	Std err	*p* > |*z*|	95% CI
PART 1				
Age	−0.26	0.08	**<.01**	−0.4, −0.1
Sex				
Female	0.25	0.18	.16	−0.1, 0.6
Ethnicity				
Non‐White Caucasian	−0.35	0.27	.20	−0.9, 0.2
Any atopy				
Yes	−0.76	0.20	**<.001**	0.4, 1.1
Annual household income				
£36,001–60,000	−0.16	0.22	.48	−0.6, 0.3
More than £60,000	−0.41	0.24	.09	−0.9, 0.1
PART 2				
Age	−22.94	26.29	.383	−74.5, 28.6
Sex				
Female	−97.42	64.82	.133	−224.5, 29.6
Ethnicity				
Non‐White Caucasian	−61.88	78.62	.431	−216.0, 92.2
Any atopic diseases				
Yes	10.25	56.70	.857	−100.9, 121.4
Annual household income				
£36,001–60,000	−196.48	92.02	**<.05**	−376.9, −16.1
More than £60,000	−253.84	91.86	**<.05**	−433.9, −73.8

*Note*: AIC = 13.9, BIC = −1523.1. Bold values are statistically significant.

## 4 DISCUSSION

Cost of illness studies aim to help highlight the economic burden of conditions for specific populations. Children with atopic diseases (i.e., food allergies, asthma, AD, allergic rhinitis) had approximately 30% higher total NHS healthcare costs compared to children without atopic disease as a result of having more visits to community and hospital healthcare services compared to those without atopic diseases although this was not statistically significant. Interestingly, 36.5% of the entire cohort had zero healthcare costs over a 12‐month period because they did not utilize any healthcare services; 75.9% of these children had no atopic diseases whilst 24.1% were children with an atopic disease.

By 7–12 years of age, from a clinical point of view, atopic diseases can stabilize or may be outgrown.^17^ However, it can also be a time when new atopic conditions are confirmed (i.e., viral induced wheeze may develop into asthma) or may start to develop (i.e., allergic rhinitis) and as seen in our cohort, continue to persist. However, there is an element where parents/families may have a better understanding of their child's illnesses and can manage them more independently without needing to seek advice from healthcare professionals. These are all factors that can have an influence on healthcare usage in children of this age group.

The atopic group had a greater mean total NHS healthcare cost compared to the children who were not atopic; the mean difference was £76.85 for total NHS healthcare cost in a year compared to non‐atopic children. Hospital inpatient admissions (0.06 admissions vs. 0.01 admissions) and specialist hospital outpatient services visits (0.27 visits vs. 0.17 visits) were higher in the children with atopy compared to those without atopic diseases, respectively. Therefore, when comparing hospital‐based costs, the atopic group had a significantly higher total cost of any hospital services compared to those without any atopic diseases (mean difference 104.33 (36.19, 172.47)). Given that the atopic diseases included food allergies, asthma, and AD, which are diseases that can require more specialist input from tertiary services for diagnosis or management, it makes sense that these children were more likely to use hospital services. There were also significant differences for children with atopic diseases in terms of the number of GP visits and practice nurse community services. This is also aligned in terms of the management of these atopic conditions as these children often require annual reviews (i.e., for asthma) or have visits for exacerbations or symptom management.

In our regression analyses, younger age and the presence of atopy were associated with being more likely to have a positive total NHS healthcare cost. For those with positive total NHS costs, lower annual household income were significantly associated with higher total NHS healthcare costs being incurred. Having an atopic disease was also associated with positive total NHS costs (coefficient −0.76, 95% CI 0.4, 1.1) (Table 3). However, in the children who had positive NHS total costs, although there was a positive association in that having atopy increased total NHS healthcare costs by £10.25, this was not statistically significant. This might reflect that we looked at the presence of any atopy rather than the number or type of atopy reported. In a study that looked at 5 different chronic conditions in children, those with asthma, diabetes and epilepsy had higher medical care expenditures compared to children without these conditions.^18^ It is likely that the more chronic conditions children have, the increased likelihood their total healthcare costs increase.

As our study focus was on atopic conditions, other chronic diseases were not accounted for in the analyses. In a study by Miller et al., children with food allergies and hypertension (i.e. the other two chronic conditions of the five included), did not have any statistically significant differences between total medical expenditures compared to children without these conditions.^18^ Different chronic conditions might incur different healthcare costs and the majority of health economic studies often focus on targeted illnesses of interest within a specific population. This might explain why we did not see a significant difference in total NHS healthcare cost for children with atopic conditions even though children with atopy had a higher mean total NHS cost.

As with many chronic diseases, it was not surprising that as children got older, they had lower total NHS healthcare costs. It is more likely that older children, particularly those in this age group of 7–12 years, have either stable atopic conditions that are under control requiring less regular healthcare professional input or that children might have outgrown some of the more common early childhood illnesses. In an Australian study which looked at healthcare utilization and costs in a cohort of children, they reported that children had more healthcare consultations and higher annual healthcare costs in the first 2 years of life compared to the next 3 years.^19^ This has also been demonstrated in a US‐based study which showed in a cohort of young children, there was a pattern of decreasing healthcare use with age likely influenced by higher rates of illness in younger children.^20^

Annual household income was also associated with total healthcare costs with total healthcare costs being significantly lower if the child was in a higher annual income household. Hayes et al., looked at factors that affected healthcare utilization and costs in Australian children and having an annual household income of less than AUD 40,000 (equivalent to approximately £20,000) was associated with increased healthcare utilization and therefore, costs (OR 3.36, 95% CI 1.3, 8.69).^19^ This does seem to reflect other studies performed in that low income is associated with increased healthcare utilization and/or costs. A Canadian study looking at children with asthma found that children from low‐income families were at higher risk of hospitalization regardless of their asthma status.^21^ However, income alone is unlikely to be the only contributor to why one seeks healthcare and various factors including the type of health conditions a person has can influence this. A US‐based study reported significant income gradients for certain health conditions (i.e., asthma, obesity, vision/hearing problems, headaches) with lower income families having greater healthcare needs.^22^ In contrast, the opposite was found for allergies (i.e., respiratory, allergic rhinitis, food, and skin allergies) in that children from higher socioeconomic status families reported worse health.^22^ This may be due to higher social class parents being more likely to seek a medical diagnosis for an allergic disorder and being able to access healthcare for these conditions but also knowledge of recognizing possible allergy symptoms to then seek a diagnosis.^22^

If we were to extrapolate our findings to the wider population, based on population statistics, there are currently approximately 4 million children in the 7–12‐year bracket^23^ so if 34% are atopic (based on the estimate in this paper) and this is multiplied by the £77 additional health care spend per child per year, the potential additional healthcare spend as a result of atopy equates to £104.7 million per year for this age band of children. This calculation applies the average additional healthcare cost per year for the 7–12 year age range and reflects the estimates derived from this EAT‐On cohort.

There are several limitations with this study. Firstly, we chose to look at the differences in cost between children with any atopic disease and children without any atopic diseases by parental report, which meant other comorbidities were not accounted for in terms of contribution to healthcare costs. We could have looked at each atopic condition individually or combinations of atopic diseases (i.e., two atopic diseases, three atopic diseases etc.); however, including two or more of these variables would have caused confounding. The numbers for each individual atopic disease were also small making it harder to look at covariates predicting costs in some of our analyses. It is also possible that other comorbidities could have impacted healthcare costs especially other chronic illnesses (i.e., type 1 diabetes sickle cell disease). However, children in either the atopic or non‐atopic group could have equally been affected by these other diseases which means this would have had less of an impact on healthcare costs between the two groups we were studying. Secondly, the healthcare costs described did involve some assumptions being made in terms of determining assignment of costs to services. All assumptions were described in Table S1 and we used the most up‐to‐date referenced sources of unit costs for a single price year when calculating healthcare costs for all the children to allow for consistency. Thirdly, HEQ looked at healthcare utilization over a 12‐month period. It is well known that participant recall of past events during data collection is at risk of recall errors (i.e., inaccurate or incomplete recollection) with studies showing under‐reporting to be more common than over‐reporting especially if they are about primary care visits.^24^ There is an inverse relationship between the length of time that subjects are asked to recall healthcare use and the accuracy of the information that is reported; so to reduce recall error the recall window can be shortened; however this comes with the trade‐off of potential information loss.^25^ Another factor we considered was the timing of when the HEQ was completed with regard to the COVID‐19 pandemic as national restrictions on leaving the house during lockdown could have affected healthcare utilization and costs. When this was accounted for in our regression analyses, there was a significant association between age and the timing of HEQ completion in relation to the COVID pandemic; so if it was included in the regression analyses age was no longer significantly related to total NHS cost. As a result, this was not included in the analyses as it was not possible to conclude whether the difference observed was due to age or the impact of COVID which likely had an impact on health care utilization. This paper focuses on the healthcare cost burden of atopic disease. Future research could estimate the healthcare burden in terms of patient and family quality of life as it is important to understand the impact of atopic disease on the financial health of families. Future research could also be undertaken to develop and validate a shorter version of the HEQ, given this study helps identify the main cost drivers.

## 5 CONCLUSION

In a general population of UK children aged 7–12 years, approximately a third had zero NHS healthcare costs over a 12‐month period. When comparing total NHS healthcare costs, children with atopic disease were more likely to incur healthcare costs and have higher costs compared to children without atopic disease, which when extrapolated equated to approximately £104.7 million more a year. This study highlights the burden of atopic disease in children that exists in a general population. Future research looking at longitudinal changes in resource use and costs in atopic children over time would be useful to understand healthcare utilization throughout childhood and allow for comparisons to be made between children of different ages and see where greater costs are incurred for atopic diseases.

## AUTHOR CONTRIBUTIONS

**Ru‐Xin Foong:** Conceptualization; data curation; methodology; investigation; formal analysis; writing – original draft; writing – review and editing. **Joanna Craven:** Data curation; software; methodology. **George Du Toit:** Writing – review and editing; investigation. **Helen A. Brough:** Supervision; writing – review and editing. **Alexandra F. Santos:** Supervision; writing – review and editing. **Gideon Lack:** Supervision; writing – review and editing. **Tracey H. Sach:** Conceptualization; methodology; data curation; writing – review and editing; supervision.

## CONFLICT OF INTEREST STATEMENT

GdT reports grants from the National Institute of Allergy and Infectious Diseases (NIAID, NIH), Food Allergy and Research Education (FARE), MRC and Asthma UK Centre, UK Dept of Health through NIHR, Action Medical Research and the National Peanut Board. Scientific Advisory Board member Aimmune. Investigator on pharma‐sponsored allergy studies (Aimmune, and DBV Technologies). Scientific advisor to Aimmune, DBV and Novartis. HAB reports grants from National Institute of Allergy and Infectious Diseases (NIAID, NIH), and speaker fees from DBV Technologies, outside of the submitted work. GL reports grants from the National Institute of Allergy and Infectious Diseases (NIAID, NIH), other from Food Allergy & Research Education (FARE), other from MRC & Asthma UK Centre, other from UK Dept of Health through NIHR, other from the National Peanut Board (NPB), other from The Davis Foundation, during the conduct of the study; shareholder in DBV Technologies, and Mighty Mission Me, personal fees from Novartis, personal fees from Sanofi‐Genzyme, personal fees from Regeneron, personal fees from ALK‐Abello, personal fees from Lurie Children's Hospital, outside the submitted work. AFS reports grants from Medical Research Council (MR/M008517/1; MC/PC/18052; MR/T032081/1), Food Allergy Research and Education (FARE), the Immune Tolerance Network/National Institute of Allergy and Infectious Diseases (NIAID, NIH), Asthma UK (AUK‐BC‐2015‐01), BBSRC, Rosetrees Trust and the NIHR through the Biomedical Research Centre (BRC) award to Guy's and St Thomas' NHS Foundation Trust, during the conduct of the study; personal fees from Thermo Scientific, Nutricia, Infomed, Novartis, Allergy Therapeutics, Buhlmann, as well as research support from Buhlmann and Thermo Fisher Scientific through a collaboration agreement with King's College London. THS reports grants from the National Institute for Health Research (NIHR206263; NIHR129926; PB‐PG‐1215‐20019; 12/67/12). THS is a steering committee member of the UK Dermatology Clinical Trials Network (July 2019–July 2025). All other authors have nothing to disclose.

## Supporting information


Data S1.
