# Supplementary material for: Investigating the health economic burden of atopic disease in children from the EAT‐On Study
Source: Pediatr Allergy Immunol. 2025 Dec 4;36(12):e70256. doi: 10.1111/pai.70256 (PMC12678843; doi:10.1111/pai.70256)
Supplement: Supplementary file 1 — Data S1. [file PAI-36-e70256-s001.zip › pai70256-sup-0001-Supinfo1@S1 Health Economics Questionnaire (HEQ).pdf]

EAT-On Study - Health Economics Questionnaire

This questionnaire has been developed as a part of the EAT-On Study and focuses on the cost of living and cost of health care for children and their families who have allergies (including food allergy, eczema, asthma, hayfever/allergic rhinitis). We want to compare this to children without allergies to see if there is a difference so it is important that families with no allergies complete the questionnaire as well. Please also complete this questionnaire even if you do not live in the UK.

For the questions that ask about your child, we would like you to answer in terms of your child that is participating on the EAT-On Study.

This questionnaire should take approximately **10 minutes** to complete. Thank you for your time and please do not hesitate to contact us if you have any questions about this or any other part of the EAT-On Study.

How many people currently live in your child's household (please include your child but exclude non-family members (i.e. live-in carers such as au pair/nanny, lodger, guests))?

- ○ 2
- ○ 3
- ○ 4
- ○ 5
- ○ 6
- ○ 7
- Q1 ○ More than 7

Q1a If more than 7 please specify how many [123] ______

Which atopic disease(s), if any, has your child been diagnosed with by a doctor?

		Yes	No
Q2a	Eczema	○	○
Q2b	Hayfever or Allergic rhinitis	○	○
Q2c	Food allergies	○	○
Q2d	Asthma	○	○
Q2e	Coeliac disease	○	○

Questions on the cost of health care visits

We are going to ask you some questions about 5 types of health care services that your child might have used in the **last 12 months**. These include:

Community (Non-hospital) Based NHS Services
NHS Hospital Outpatient Services
Accident & Emergency NHS Services
Inpatient NHS hospital stays
Private Sector health care Services

Community (Non-Hospital) Based NHS Services

These services refer to any NHS services that your child has used outside of the hospital setting. These include visits to your GP, any walk-in-centre services in the community, visits with any other health care professionals outside of the hospital setting.

In the last **12 months,** has your child visited any **community (non-hospital) based NHS services** (excluding hospital outpatient appointments and hospital admissions)?

		Yes	No
Q3a	General Practitioner (GP)	○	○
Q3b	GP Practice nurse	○	○
Q3c	Community physiotherapist	○	○
Q3d	Community occupational therapist	○	○
Q3e	Community dietician	○	○
Q3f	Psychology or counselling services	○	○
Q3g	Other community health care professionals	○	○

Q4 Please specify how many **other community health care professionals** your child saw in the last 12 months?

○ 1
○ 2

Q4a	Type of community health care professional 1	
Q4b	Number of visits	123
Q4c	Type of community health care professional 2	
Q4d	Number of visits	123

How many visits has your child had in the community with your:

		1	2	3	4	5	6	7	8	9	10	More than 10
Q5a	GP	○	○	○	○	○	○	○	○	○	○	○
Q5b	GP practice nurse	○	○	○	○	○	○	○	○	○	○	○
Q5c	Community physiotherapist	○	○	○	○	○	○	○	○	○	○	○
Q5d	Community occupational therapist	○	○	○	○	○	○	○	○	○	○	○
Q5e	Community dietician	○	○	○	○	○	○	○	○	○	○	○
Q5f	Psychology or counselling services	○	○	○	○	○	○	○	○	○	○	○

Q5g If more than 10 with GP, please state how many 123 [ ]

Q5h If more than 10 with GP practice nurse, please state how many 123 [ ]

Q5i If more than 10 with community physiotherapist, please state how many 123 [ ]

Q5j If more than 10 with community occupational therapist, please state how many 123 [ ]

Q5k If more than 10 with community dietician, please state how many 123 [ ]

Q5l If more than 10 with psychology or counselling services, please state how many 123 [ ]

What was the main reason for your child's **psychology or counselling visits**?

Q6 --Click Here--

- Anxiety
- Behavioural issues
- Depression
- Allergies
- Other

Q6a If Other, please explain here [ ]

How many visits to the **GP** were related to your child's **food allergies**?

--Click Here--

None
1
2
3
4
5
6
7
8
9
10
Q7 More than 10

Q7a If more than 10, please state how many

How many visits to the **GP** were related to your child's **asthma**?

--Click Here--

None
1
2
3
4
5
6
7
8
9
10
Q8 More than 10

Q8a If more than 10, please state how many

How many visits to the **GP** were related to your child's **eczema**?

--Click Here--

None
1
2
3
4
5
6
7
8
9
10
Q9 More than 10

Q9a If more than 10, please state how many

How many visits to the **GP nurse** were related to your child's **food allergies**?

--Click Here--

None
1
2
3
4
5
6
7
8
9
10
Q10 More than 10

Q10a If more than 10, please state how many

How many visits to the **GP nurse** were related to your child's **asthma**?

--Click Here--

None
1
2
3
4
5
6
7
8
9
10
Q11 More than 10

Q11a If more than 10, please state how many

How many visits to the **GP nurse** were related to your child's **eczema**?

--Click Here--

None
1
2
3
4
5
6
7
8
9
10
Q12 More than 10

Q12a If more than 10, please state how many

How many visits to the **dietician** were related to your child's **food allergies**?

--Click Here--

- None
- 1
- 2
- 3
- 4
- 5
- 6
- 7
- 8
- 9
- 10
- More than 10

Q13

Q13a If more than 10, please state how many

In the **last 12 months**, was your child given any **medical prescriptions** by any professional in **community (non hospital) based NHS Services** (i.e. prescription issued and collected in the community)?

- Yes
- No

Q14

How many medical prescriptions has your child received from any professional in **community (non hospital) based NHS Services**?

--Click Here--

- 1
- 2
- 3
- 4
- 5
- 6
- 7
- 8
- 9
- 10
- More than 10

Q15

Q15a If more than 10, please state how many

123

How many of these **community (non hospital) based NHS prescriptions** were related to an atopic disease (i.e. eczema, asthma, hayfever or allergic rhinitis, food allergies)

--Click Here--

- 0
- 1
- 2
- 3
- 4
- 5
- 6
- 7
- 8
- 9
- 10
- More than 10

Q16

Q16a If more than 10, please state how many [123] ______

How many of these prescriptions were for one or more autoadrenaline injector devices (i.e. Epipen, Jext, Emerade)?

--Click Here--

- None
- 1
- 2
- 3
- 4
- 5
- 6
- 7
- 8
- 9
- 10
- More than 10

Q17

Q17a If more than 10, please state how many ______

NHS Hospital outpatient services

These services refer to any NHS appointments/visits that your child has had with a health care professional that has taken place within a hospital setting. These do not include any inpatient admissions or attendance at any emergency (A&E) services.

In the last **12 months**, has your child visited any of the following health care professionals for their health in **NHS Hospital Outpatient Services**?

		Yes	No
Q18a	Paediatric Allergy outpatient clinic	○	○
Q18b	Paediatric Respiratory outpatient clinic	○	○

Q18c	Paediatric Dermatology outpatient clinic	○	○
Q18d	Paediatric Gastroenterology outpatient clinic	○	○
Q18e	Paediatric Ears/Nose/Throat outpatient clinic	○	○
Q18f	General paediatrics outpatient clinic	○	○
Q18g	Hospital paediatric specialist nurse	○	○
Q18h	Hospital paediatric dietician	○	○
Q18i	Hospital paediatric physiotherapist	○	○
Q18j	Hospital paediatric occupational therapist	○	○
Q18k	Hospital paediatric psychology or counselling therapist	○	○
Q18l	Other health care professional (in hospital)	○	○

Q19 Please specify how many **other hospital health care professionals** your child saw in the last 12 months?

○ 1
○ 2

Q19a	Type of other hospital health care professional 1	
Q19b	Number of visits	123
Q19c	Type of other hospital health care professional 2	
Q19d	Number of visits	123

How many visits has your child had in the last 12 months with or in:

		1	2	3	4	5	6	7	8	9	10	More than 10
Q20a	Paediatric Allergy outpatient clinic	○	○	○	○	○	○	○	○	○	○	○
Q20b	Paediatric Respiratory outpatient clinic	○	○	○	○	○	○	○	○	○	○	○
Q20c	Paediatric Dermatology outpatient clinic	○	○	○	○	○	○	○	○	○	○	○
Q20d	Paediatric Gastroenterology outpatient clinic	○	○	○	○	○	○	○	○	○	○	○
Q20e	Paediatric Ears/Nose/Throat outpatient clinic	○	○	○	○	○	○	○	○	○	○	○
Q20f	General paediatrics outpatient clinic	○	○	○	○	○	○	○	○	○	○	○
Q20g	Hospital paediatric specialist nurse	○	○	○	○	○	○	○	○	○	○	○
Q20h	Hospital paediatric dietician	○	○	○	○	○	○	○	○	○	○	○
Q20i	Hospital paediatric physiotherapist	○	○	○	○	○	○	○	○	○	○	○

Q20j	Hospital paediatric occupational therapist	○	○	○	○	○	○	○	○	○	○	○
Q20k	Hospital paediatric psychology or counselling therapist	○	○	○	○	○	○	○	○	○	○	○

Q20l If more than 10 in Paediatric Allergy, please state how many 123 [ ]

Q20m If more than 10 in Paediatric Respiratory, please state how many 123 [ ]

Q20n If more than 10 in Paediatric Dermatology, please state how many 123 [ ]

Q20o If more than 10 in Paediatric Gastroenterology, please state how many 123 [ ]

Q20p If more than 10 in Paediatric Ears/Nose/Throat, please state how many 123 [ ]

Q20q If more than 10 in Paediatric General paediatrics, please state how many 123 [ ]

Q20r If more than 10 in hospital paediatric specialist nurse, please state how many 123 [ ]

Q20s If more than 10 in hospital paediatric dietician, please state how many 123 [ ]

Q20t If more than 10 in hospital paediatric physiotherapist, please state how many 123 [ ]

Q20u If more than 10 in hospital paediatric occupational therapist, please state how many 123 [ ]

Q20v If more than 10 in paediatric psychology or counselling, please state how many 123 [ ]

Q21 What was the main reason for your child's psychology or counselling visits?

--Click Here--

- Anxiety
- Behavioural issues
- Depression
- Allergies
- Other

Q21a If Other, please explain here [ ]

How many visits to the **General Paediatric clinic** were related to your child's **food allergies**?

--Click Here--

None
1
2
3
4
5
6
7
8
9
10
Q22 More than 10

Q22a If more than 10, please state how many

How many visits to **General Paediatric clinic** were related to your child's **asthma**?

--Click Here--

None
1
2
3
4
5
6
7
8
9
10
Q23 More than 10

Q23a If more than 10, please state how many

How many visits to **General Paediatric clinic** were related to your child's **eczema**?

--Click Here--

None
1
2
3
4
5
6
7
8
9
10
Q24 More than 10

Q24a If more than 10, please state how many

How many visits to the **hospital specialist nurse** were related to your child's **food allergies**?

--Click Here--

None
1
2
3
4
5
6
7
8
9
10
Q25 More than 10

Q25a If more than 10, please state how many

How many visits to the **hospital specialist nurse** were related to your child's **asthma**?

--Click Here--

None
1
2
3
4
5
6
7
8
9
10
Q26 More than 10

Q26a If more than 10, please state how many

How many visits to the **hospital specialist nurse** were related to your child's **eczema**?

--Click Here--

None
1
2
3
4
5
6
7
8
9
10
Q27 More than 10

Q27a If more than 10, please state how many

How many visits to the **hospital dietician** were related to your child's **food allergies**?

Q28 --Click Here--
- None
- 1
- 2
- 3
- 4
- 5
- 6
- 7
- 8
- 9
- 10
- More than 10

Q28a If more than 10, please state how many

Has your child been discharged from one of the following services but is due to be seen again for follow up **within the next 3 years** by a health care professional for their health in **NHS Hospital Outpatient Services**?

		Yes	No
Q29a	Paediatric Allergy outpatient clinic	○	○
Q29b	Paediatric Respiratory outpatient clinic	○	○
Q29c	Paediatric Dermatology outpatient clinic	○	○
Q29d	Paediatric Gastroenterology outpatient clinic	○	○
Q29e	Paediatric Ears/Nose/Throat outpatient clinic	○	○
Q29f	General paediatrics outpatient clinic	○	○
Q29g	Hospital paediatric specialist nurse	○	○
Q29h	Hospital paediatric dietician	○	○
Q29i	Hospital paediatric physiotherapist	○	○
Q29j	Hospital paediatric occupational therapist	○	○
Q29k	Hospital paediatric psychology or counselling therapist	○	○

In the **last 12 months**, was your child given any **medical prescriptions** by **any heath care professional in Paediatric Hospital Outpatient Services** (i.e. prescription issued and then collected from a hospital pharmacy)?

Q29
- ○ Yes
- ○ No

In the **last 12 months** how many **medical prescriptions** has your child received from **any health care professional in Paediatric Hospital Outpatient Services?**

--Click Here--

1
2
3
4
5
6
7
8
9
10
Q30 More than 10

Q30a If more than 10, please state how many [123] ______

How many of these **hospital medical prescriptions** were related to an atopic disease (i.e. eczema, asthma, hayfever or allergic rhinitis, food allergies)?

--Click Here--

None
1
2
3
4
5
6
7
8
9
10
Q31 More than 10

Q31a If more than 10, please state how many [123] ______

How many of these prescriptions were for one or more autoadrenaline injector devices (i.e. Epipen, Jext, Emerade)?

--Click Here--

None
1
2
3
4
5
6
7
8
9
10
Q32 More than 10

Q32a If more than 10, please state how many

NHS Accident & Emergency Services

These services refer to any attendances your child might have had to an A&E Department. This includes your child being seen in a minor accident unit, a hospital run GP service or by a nurse or doctor in the main children A&E department.

Q33 In the last **12 months**, did your child need to visit any **NHS Accident and Emergency Departments**?

- Yes
- No

Q34 How many visits has your child had to **NHS A&E Department(s)** in the last 12 months?

--Click Here--

1
2
3
4
5
6
7
8
9
10
More than 10

Q34a If more than 10, please state how many 123

Q35 How many of these visits were related to their atopic disease (i.e. eczema, asthma, hayfever or allergic rhinitis, food allergies)?

--Click Here--

None
1
2
3
4
5
6
7
8
9
10
More than 10

Q35a If more than 10, please state how many 123

How many of these visits were related to an allergic reaction including anaphylaxis?

--Click Here--

None
1
2
3
4
5
6
7
8
9
10
Q36 More than 10

Q36a If more than 10, please state how many

How many of these visits were related to asthma (i.e. asthma exacerbation)?

--Click Here--

None
1
2
3
4
5
6
7
8
9
10
Q37 More than 10

Q37a If more than 10, please state how many

How many of these visits were related to eczema (i.e. eczema flare or infected eczema)?

--Click Here--

None
1
2
3
4
5
6
7
8
9
10
Q38 More than 10

Q38a If more than 10, please state how many

In the **last 12 months**, how many **medical prescriptions** was your child given from an A&E department?

--Click Here--

None
1
2
3
4
5
6
7
8
9
10
Q39 More than 10

Q39a If more than 10, please state how many 123

How many of these **A&E NHS medical prescriptions** were related to an atopic disease (i.e. eczema, asthma, hayfever or allergic rhinitis, food allergies)?

--Click Here--

None
1
2
3
4
5
6
7
8
9
10
Q40 More than 10

Q40a If more than 10, please state how many 123

How many of these medical prescriptions were for one or more autoadrenaline injector devices (i.e. Epipen, Jext, Emerade)?

--Click Here--

Q41

- None
- 1
- 2
- 3
- 4
- 5
- 6
- 7
- 8
- 9
- 10
- More than 10

Q41a If more than 10, please state how many

Inpatient NHS Hospital Stays

This refers to <u>any admissions</u> your child has had to any hospital in the last 12 months. This includes day-case and/or overnight admissions for procedures/surgeries/medical care that may have been planned or due to emergencies. Please answer the questions below to the best of your ability.

In the **last 12 months**, has your child had any admissions to a **NHS hospital as an inpatient**?

Q42

- Yes
- No

How many of these admissions were:

		0	1	2	3	4	More than 4
Q43a	Planned admission(s)	○	○	○	○	○	○
Q43b	Emergency admission(s)	○	○	○	○	○	○

Q43c If more than 4 <u>planned admissions</u>, please specify how many 123

Q43d If more than 4 <u>emergency admissions</u> please specify how many 123

How many nights did your child stay in hospital during each planned hospital admission?

		Day only (did not need to stay ov ernight)	1 night	2 nights	3 nights	4 nights	5 nights	6 nights	7 nights	More than 7 nights
Q44a	Planned admission 1	○	○	○	○	○	○	○	○	○

Q44b	Planned admission 2	○	○	○	○	○	○	○	○	○
Q44c	Planned admission 3	○	○	○	○	○	○	○	○	○
Q44d	Planned admission 4	○	○	○	○	○	○	○	○	○

Q44e If more than 7 nights (planned admission 1), please state how many 123 [ ]

Q44f If more than 7 nights (planned admission 2), please state how many 123 [ ]

Q44g If more than 7 nights (planned admission 3), please state how many 123 [ ]

Q44h If more than 7 nights (planned admission 4), please state how many 123 [ ]

Q44i If they had more than 4 planned hospital admissions, please tell us how many nights they stayed for each additional visit [ ]

Were any of these planned admissions related to an atopic disease (i.e. eczema, asthma, hayfever or allergic rhinitis, food allergies)?

		Yes	No
Q45a	Planned admission 1	○	○
Q45b	Planned admission 2	○	○
Q45c	Planned admission 3	○	○
Q45d	Planned admission 4	○	○

Q45e If they had more than 4 planned hospital admissions, please tell us how many of the additional admissions were related to atopic disease? [ ]

Which type of ward did your child stay in during each planned admission?

		Children short stay unit or overnight stay in unit attached to an A&E department	Children inpatient ward	Paediatric intensive care unit or high dependency unit	Unsure or don't remember
Q46a	Planned admission 1	○	○	○	○
Q46b	Planned admission 2	○	○	○	○
Q46c	Planned admission 3	○	○	○	○
Q46d	Planned admission 4	○	○	○	○

Q46e If they had more than 4 planned hospital admissions, please tell us where they stayed in hospital for each admission

Q46f Please name the ward and/or hospital for planned admission 1

Q46g Please name the ward and/or hospital for planned admission 2

Q46h Please name the ward and/or hospital for planned admission 3

Q46i Please name the ward and/or hospital for planned admission 4

How many nights did your child stay in hospital during each emergency hospital admission?

		Day only (did not need to stay ov ernight)	1 night	2 nights	3 nights	4 nights	5 nights	6 nights	7 nights	More than 7 nights
Q47a	Emergency admission 1	○	○	○	○	○	○	○	○	○
Q47b	Emergency admission 2	○	○	○	○	○	○	○	○	○
Q47c	Emergency admission 3	○	○	○	○	○	○	○	○	○
Q47d	Emergency admission 4	○	○	○	○	○	○	○	○	○

Q47e If more than 7 nights (emergency admission 1), please state how many 123

Q47f If more than 7 nights (emergency admission 2), please state how many 123

Q47g If more than 7 nights (emergency admission 3), please state how many 123

Q47h If more than 7 nights (emergency admission 4), please state how many 123

Q47i If they had more than 4 emergency hospital admissions, please tell us how many nights they stayed for each additional visit

Were any of these emergency admissions related to an atopic disease (i.e. eczema, asthma, hayfever or allergic rhinitis, food allergies)?

		Yes	No
Q48a	Emergency admission 1	○	○
Q48b	Emergency admission 2	○	○
Q48c	Emergency admission 3	○	○
Q48d	Emergency admission 4	○	○

Q48e If they had more than 4 emergency hospital admissions, please tell us how many of the additional admissions were related to atopic disease?

Which type of ward did your child stay in during each emergency hospital admission?

		Children short stay unit or overnight stay in unit attached to an A&E department	Children inpatient ward	Paediatric intensive care unit or high dependency unit	Unsure or don't remember
Q49a	Emergency admission 1	○	○	○	○
Q49b	Emergency admission 2	○	○	○	○
Q49c	Emergency admission 3	○	○	○	○
Q49d	Emergency admission 4	○	○	○	○

Q49e If they had more than 4 emergency hospital admissions, please tell us where they stayed in hospital for each admission

Q49f Please name the ward and/or hospital for emergency admission 1

Q49g Please name the ward and/or hospital for emergency admission 2

Q49h Please name the ward and/or hospital for emergency admission 3

Q49i Please name the ward and/or hospital for emergency admission 4

Private health care services

These services refer to any health care professional that your child may have seen that you have arranged and paid for privately. This includes visits covered by private health care insurance.

In the last **12 months**, has your child visited any health care professional in the **Private health care Sector** (i.e.those that you pay for yourself or via private health care insurance)?

Q50
- ◯ Yes
- ◯ No

How did you pay for the **Private health care** your child received?

Q51
- ◯ Self-funded (no insurance, out of pocket cost)
- ◯ Self-funded private insurance
- ◯ Private insurance (through parent's job)

Q51a Please describe what type of private insurance cover you have (name of insurance company, type of policy if known) ________

Q51b If relevant, please specify how much you pay **per month** for private health care insurance (round to the nearest £) 123 ________

Which health care professional did your child see in the **Private health care Sector**?

		Yes	No
Q52a	Private Paediatric Allergy Consultant	◯	◯
Q52b	Private Paediatric Respiratory Consultant	◯	◯
Q52c	Private Paediatric Dermatology Consultant	◯	◯
Q52d	Private Paediatric Gastroenterology Consultant	◯	◯
Q52e	Private Paediatric Ears/Nose/Throat Consultant	◯	◯
Q52f	Private General paediatrian	◯	◯
Q52g	Private paediatric specialist nurse	◯	◯
Q52h	Private Paediatric dietician	◯	◯
Q52i	Private Paediatric physiotherapist	◯	◯
Q52j	Private Paediatric occupational therapist	◯	◯
Q52k	Private Paediatric psychology or counselling therapist	◯	◯
Q52l	Other private health care professional	◯	◯

Please specify how many **other private health care professional** your child saw in the last 12 months?

Q53
- ◯ 1
- ◯ 2

Q53a Type of other private health care professional 1 [ ]

Q53b Number of visits 123 [ ]

Q53c Type of other private health care professional 2 [ ]

Q53d Number of visits 123 [ ]

What was the average cost of a single consultation with the **other Private paediatric health care professional 1**?

--Click Here--

- Less than £100
- £101-£150
- £151-£200
- £201-£250
- £251-£300
- £301-£350
- £351-£400
- £401-£450
- £451-£500
- Q54 More than £500

Q54a If more than £500, please state how much (£) 123 [ ]

What was the average cost of a single consultation with the **other Private paediatric health care professional 2**?

--Click Here--

- Less than £100
- £101-£150
- £151-£200
- £201-£250
- £251-£300
- £301-£350
- £351-£400
- £401-£450
- £451-£500
- Q55 More than £500

Q55a If more than £500, please state how much (£) 123 [ ]

How many visits has your child had in the last 12 months with:

		1	2	3	4	5	6	7	8	9	10	More than 10
Q56a	Private Paediatric Allergy Consultant	○	○	○	○	○	○	○	○	○	○	○
Q56b	Private Paediatric Respiratory Consultant	○	○	○	○	○	○	○	○	○	○	○
Q56c	Private Paediatric Dermatology Consultant	○	○	○	○	○	○	○	○	○	○	○

Q56d	Private Paediatric Gastroenterology Consultant	○	○	○	○	○	○	○	○	○	○	○
Q56e	Private Paediatric Ears/Nose/Throat Consultant	○	○	○	○	○	○	○	○	○	○	○
Q56f	Private General paediatrian	○	○	○	○	○	○	○	○	○	○	○
Q56g	Private paediatric specialist nurse	○	○	○	○	○	○	○	○	○	○	○
Q56h	Private Paediatric dietician	○	○	○	○	○	○	○	○	○	○	○
Q56i	Private Paediatric physiotherapist	○	○	○	○	○	○	○	○	○	○	○
Q56j	Private Paediatric occupational therapist	○	○	○	○	○	○	○	○	○	○	○
Q56k	Private Paediatric psychology or counselling therapist	○	○	○	○	○	○	○	○	○	○	○

Q56l If more than 10 with Private Paediatric Allergy Consultant, please state how many 123 ________

Q56m If more than 10 with Private Paediatric Respiratory Consultant, please state how many 123 ________

Q56n If more than 10 with Private Paediatric Dermatology Consultant, please state how many 123 ________

Q56o If more than 10 with Private Paediatric Gastroenterology Consultant, please state how many 123 ________

Q56p If more than 10 with Private Paediatric Ears/Nose/Throat Consultant, please state how many 123 ________

Q56q If more than 10 with Private General Paediatrian, please state how many 123 ________

Q56r If more than 10 with Private paediatric specialist nurse, please state how many 123 ________

Q56s If more than 10 with Private paediatric dietician, please state how many 123 ________

Q56t If more than 10 with Private paediatric physiotherapist, please state how many 123 ________

Q56u If more than 10 with Private paediatric occupational therapist, please state how many

123 [ ]

Q56v If more than 10 with Private psychology or counselling therapist, please state how many

123 [ ]

What was the average cost of a single consultation with a:

		Less than £100	£101-150	£151-200	£201-250	£251-300	£301-350	£351-£400	£401-450	£451-500	More than £500
Q57a	Private Paediatric Allergy Consultant	○	○	○	○	○	○	○	○	○	○
Q57b	Private Paediatric Respiratory Consultant	○	○	○	○	○	○	○	○	○	○
Q57c	Private Paediatric Dermatology Consultant	○	○	○	○	○	○	○	○	○	○
Q57d	Private Paediatric Gastroenterology Consultant	○	○	○	○	○	○	○	○	○	○
Q57e	Private Paediatric Ears/Nose/Throat Consultant	○	○	○	○	○	○	○	○	○	○
Q57f	Private General paediatrian	○	○	○	○	○	○	○	○	○	○
Q57g	Private paediatric specialist nurse	○	○	○	○	○	○	○	○	○	○
Q57h	Private Paediatric dietician	○	○	○	○	○	○	○	○	○	○
Q57i	Private Paediatric physiotherapist	○	○	○	○	○	○	○	○	○	○
Q57j	Private Paediatric occupational therapist	○	○	○	○	○	○	○	○	○	○
Q57k	Private Paediatric psychology or counselling therapist	○	○	○	○	○	○	○	○	○	○

Q57l If more than £500 with Private Paediatric Allergy Consultant, please state how much

123 [ ]

Q57m If more than £500 with Private Paediatric Respiratory Consultant, please state how much

123 [ ]

Q57n If more than £500 with Private Paediatric Dermatology Consultant, please state how much

123 [ ]

Q57o If more than £500 with Private Paediatric Gastroenterology Consultant, please state how much

123 [ ]

Q57p If more than £500 with Private Paediatric Ears/Nose/Throat Consultant, please state how much

123 [ ]

Q57q If more than £500 with Private General Paediatrian, please state how much

123 [ ]

Q57r If more than £500 with Private paediatric specialist nurse, please state how much

123 [ ]

Q57s If more than £500 with Private paediatric dietician, please state how much

123 [ ]

Q57t If more than £500 with Private Paediatric physiotherapist, please state how much

123 [ ]

Q57u If more than £500 with Private Paediatric occupational therapist, please state how much

123 [ ]

Q57v If more than £500 with Private Paediatric psychology or counselling therapist, please state how much

123 [ ]

What was the main reason for your child's **psychology or counselling** visits?

--Click Here--

- Anxiety
- Behavioural issues
- Depression
- Allergies
- Other

Q58

Q58a If Other, please explain here [ ]

What was your <u>main reason</u> for choosing to see a private health care professional?

- ○ Shorter waiting time (i.e. compared to NHS referral)
- ○ Expertise of the health care professional
- ○ Second opinion
- ○ Lack of specialist/health care professional in local area
- ○ Other (i.e. specialist treatment - food challenge)

Q59

Q59a If Other, please explain here [ ]

In the **last 12 months**, was your child given any **medical prescriptions** by **any professional in the Private health care sector**?

- ○ Yes
- ○ No

Q60

How many **private medical prescriptions** has your child received?

--Click Here--

1
2
3
4
5
6
7
8
9
10
Q61 More than 10

Q61a If more than 10, please state how many [123] [ ]

How many of these **private medical prescriptions** were related to an atopic disease (i.e. eczema, asthma, hayfever or allergic rhinitis, food allergies)?

--Click Here--

None
1
2
3
4
5
6
7
8
9
10
Q62 More than 10

Q62a If more than 10, please state how many [123] [ ]

How many of these medical prescriptions were for one or more autoadrenaline injector devices (i.e. Epipen, Jext, Emerade)?

--Click Here--

None
1
2
3
4
5
6
7
8
9
10
Q63 More than 10

Q63a If more than 10, please state how many

How much did you pay on average for <u>each</u> private medical prescription?

--Click Here--

- Less than £10
- £11-20
- £21-30
- £31-40
- £41-50
- More than £50

Q64

Q64a If more than £50, please state how much

Has your child had any **admissions to a private hospital** as an inpatient (i.e. day-case or overnight admissions for procedures or surgeries or medical care)?

- Yes
- No

Q65

How many private hospital admissions did they have in the last 12 months?

		1	2	3	4	More than 4
Q66a	Number of admission(s)	○	○	○	○	○

Q66b If more than 4 <u>private admissions</u>, please specify how many

How many nights did your child stay in hospital for each private hospital admission?

		Day only (did not need to stay ov ernight)	1 night	2 nights	3 nights	4 nights	5 nights	6 nights	7 nights	More than 7 nights
Q67a	Private admission 1	○	○	○	○	○	○	○	○	○
Q67b	Private admission 2	○	○	○	○	○	○	○	○	○
Q67c	Private admission 3	○	○	○	○	○	○	○	○	○
Q67d	Private admission 4	○	○	○	○	○	○	○	○	○

Q67e If more than 7 nights (private admission 1), please state how many

Q67f If more than 7 nights (private admission 2), please state how many

Q67g If more than 7 nights (private admission 3), please state how many

Q67h If more than 7 nights (private admission 4), please state how many

123 [ ]

Q67i If they had more than 4 private hospital admissions, please tell us how many nights they stayed for each additional visit

[ ]

Were any private hospital admissions related to an atopic disease (i.e. eczema, asthma, hayfever or allergic rhinitis, food allergies)?

		Yes	No
Q68a	Private admission 1	○	○
Q68b	Private admission 2	○	○
Q68c	Private admission 3	○	○
Q68d	Private admission 4	○	○

Q68e If they had more than 4 private hospital admissions, please tell us how many of the additional admissions were related to atopic disease?

[ ]

What was the average cost per stay during each private hospital admission?

Q69a Private admission 1 cost (£) 123 [ ]

Q69b Private admission 2 cost (£) 123 [ ]

Q69c Private admission 3 cost (£) 123 [ ]

Q69d Private admission 4 cost (£) 123 [ ]

Q69e If they had more than 4 private hospital admissions, please tell us how much you paid (on average in £) for each private hospital admission

[ ]

The following questions look at any <u>time off work</u> that anyone who helps to care for your child has had to take as a result of your child being ill and/or for appointments to see any health care professionals. It also looks at days your child has missed from school.

In the last **12 months**, have you, your partner or a friend or relative had to **take time off work** as a result of your child experiencing any ill health (i.e. to look after them at home or to accompany them to medical appointments, hospitalizations)?

- ◯ Yes
- ◯ No

Q70

Who had to take **time off work** as a result of your child experiencing ill health or needing to attend medical appointments (please tick all those that apply)?

- ☐ You
- ☐ Your partner
- ☐ Other friend or relative

Q71

Was your time off work paid or unpaid leave (please tick all those that apply)?

- ☐ Paid leave
- ☐ Unpaid leave

Q72

If **you** had to take paid leave, please specify the number of hours of leave that you had to take off in the last 12 months. (For example, you may have only required a few hours off work to attend an appointment (i.e. 3.5 hours). If you took a full day off work, calculate how many hours of that would have been of a working day - i.e. 9 working hours rather than 24 hours).

Q73a Total number of hours 123 [ ]

Q73b Number of hours related to atopic disease (i.e. eczema, asthma, hayfever or allergic rhinitis, food allergies) 123 [ ]

If **you** had to take unpaid leave, please specify the number of hours of leave that you had to take off in the last 12 months. (For example, you may have only required a few hours off work to attend an appointment (i.e. 3.5 hours). If you took a full day off work, calculate how many hours of that would have been of a working day - i.e. 9 working hours rather than 24 hours).

Q74a Total number of hours 123 [ ]

Q74b Number of hours related to atopic disease (i.e. eczema, asthma, hayfever or allergic rhinitis, food allergies) 123 [ ]

If **your partner** had to take time off work, was this paid or unpaid leave?

- ☐ Paid leave
- ☐ Unpaid leave

Q75

If **your partner** had to take paid leave, please specify the number of hours of leave that they had to take off in the last 12 months. (For example, they may have only required a few hours off work to attend an appointment (i.e. 3.5 hours). If they took a full day off work, calculate how many hours of that would have been of a working day - i.e. 9 working hours rather than 24 hours).

Q76a Total number of hours [123] ______

Q76b Number of hours related to atopic disease (i.e. eczema, asthma, hayfever or allergic rhinitis, food allergies) [123] ______

If **your partner** had to take unpaid leave, please specify the number of hours of leave that they had to take off in the last 12 months. (For example, they may have only required a few hours off work to attend an appointment (i.e. 3.5 hours). If they took a full day off work, calculate how many hours of that would have been of a working day - i.e. 9 working hours rather than 24 hours).

Q77a Total number of hours [123] ______

Q77b Number of hours related to atopic disease (i.e. eczema, asthma, hayfever or allergic rhinitis, food allergies) [123] ______

If **a friend or relative** had to take time off work, was this paid or unpaid leave?

Q78
- ☐ Paid leave
- ☐ Unpaid leave

If **a friend or relative** had to take paid leave, please specify the number of hours of leave that they had to take off in the last 12 months. (For example, they may have only required a few hours off work to attend an appointment (i.e. 3.5 hours). If they took a full day off work, calculate how many hours of that would have been of a working day - i.e. 9 working hours rather than 24 hours).

Q79a Total number of hours [123] ______

Q79b Number of hours related to atopic disease (i.e. eczema, asthma, hayfever or allergic rhinitis, food allergies) [123] ______

If **a friend or relative** had to take unpaid leave, please specify the number of hours of leave that they had to take off in the last 12 months. (For example, they may have only required a few hours off work to attend an appointment (i.e. 3.5 hours). If they took a full day off work, calculate how many hours of that would have been of a working day - i.e. 9 working hours rather than 24 hours).

Q80a Total number of hours [123] ______

Q80b Number of hours related to atopic disease (i.e. eczema, asthma, hayfever or allergic rhinitis, food allergies) [123] ______

Do you have any care arrangements for your child that you do not pay for? (i.e. stay at home parent, grandparents (i.e. excludes paid child minder or au pair/nanny)

- Yes
- No

Q81

Q81a Please describe what arrangements you have

In the last **12 months**, has your child had to have time off school due to illness or for a medical appointment?

- Yes
- No

Q82

How many days off school has your child had due to illness or for a medical appointment?

--Click Here--

1
2
3
4
5
6
7
8
9
10
11
12
13
14
More than 14

Q83

Q83a If more than 14, please state how many

123

How many of these days off school were related due to atopic disease (i.e. eczema, asthma, hayfever or allergic rhinitis, food allergies)?

--Click Here--

0
1
2
3
4
5
6
7
8
9
10
11
12
13
14
Q84 More than 14

Q84a If more than 14, please state how many [123]

The following questions relate to general costs of living

How many adults in your household are earning an income (please exclude non-family members (i.e. live-in carers such as au pair/nanny, lodger, guests))?

- None
- 1
- 2
- Q85 More than 2

Q85a Please specify how many [123]

What is your total **net household wage per month**? (Net = income after tax and other deductions)

--Click Here--

Less than £1000
£1001-£2000
£2001-£3000
£3001-£4000
£4001-£5000
£5001-£6000
£6001-£7000
£7001-£8000
£8001-£9000
£9001-£10000
Q86 More than £10000

Q86a If more than £10000, please specify how much (£) [123]

What is your total **net household wage per month**? Please add together all wages/income if there are multiple household earners. (Net = income after tax and other deductions)

--Click Here--

- Less than £2000
- £2001-£3000
- £3001-£4000
- £4001-£5000
- £5001-£6000
- £6001-£7000
- £7001-£8000
- £8001-£9000
- £9001-£10000
- £10001-£11000
- £11001-£12000
- £12001-£13000
- £13001-£14000
- £14001-£15000
- £15001-£16000
- £16001-£17000
- £17001-£18000
- £18001-£19000
- £19001-£20000
- More than £20000

Q87

Q87a If more than £20000, please specify how much (£)

Do you receive any disability living allowances for your child?

- Yes
- No

Q88

Q88a If yes, please state how much you receive **per month** (£): 123

Is the disability living allowance you receive for your child related to an atopic disease (i.e. eczema, asthma, hayfever or allergic rhinitis, food allergies)?

- Yes
- No

Q89

On average, how much is spent **each week** on food shopping for your household?

--Click Here--

- Less than £50
- £50
- £60
- £70
- £80
- £90
- £100
- £110
- £120
- £130
- £140
- £150
- £160
- £170
- £180
- £190
- £200
- Q90 More than £200

Q90a If more than £200, please specify how much (£)

On average, how many foods on your weekly shopping list are specialty items in relation to your child's allergies (i.e. if you buy gluten-free bread and gluten-free pasta, count each item separately)?

--Click Here--

- 0
- 1
- 2
- 3
- 4
- 5
- 6
- 7
- 8
- 9
- 10
- Q91 More than 10

Q91a If more than 10, please specify how many

123

On average, how much money do you spend **each week** on food shopping for specialty foods specifically due to your child's allergies (i.e. gluten-free, dairy-free, egg-free, nut-free foods etc)?

Q92 --Click Here--
- Less than £20
- £20
- £30
- £40
- £50
- £60
- £70
- £80
- £90
- £100
- More than £100

Q92a If more than £100, please specify how much (£) [123] ______

In the last **1 month**, how many meals has your child eaten outside of your home?

		0	1	2	3	4	More than 4
Q93a	Restaurant or Cafe	○	○	○	○	○	○
Q93b	Takeaway or Delivery food	○	○	○	○	○	○
Q93c	Relative or friend's home	○	○	○	○	○	○

Q93d Please specify if more than 4 meals (restaurant/cafe) [123] ______

Q93e Please specify if more than 4 times (takeaway/delivery) [123] ______

Q93f Please specify if more than 4 times (relative/friend's home) [123] ______

What is the average cost of <u>your child's meal</u> that you would spend outside in **a restaurant or cafe**?

Q94 --Click Here--
- Less than £10
- £10
- £15
- £20
- £25
- £30
- £35
- £40
- £45
- £50
- More than £50

Q94a If more than £50, please specify how much (£) [123] ______

What is the average cost of <u>your child's meal</u> that you would spend on **takeaway or delivery food**?

--Click Here--

- Less than £10
- £10
- £15
- £20
- £25
- £30
- £35
- £40
- £45
- £50
- More than £50

Q95

Q95a If more than £50, please specify how much (£) [123] [ ]

In the last **12 months** have you spent your own money on the following products specifically related to your child's atopic disease (i.e. asthma, food allergies, hayfever or allergic rhinitis)?

- ☐ None
- ☐ Over the counter medications (i.e antihistamines, nasal steroid sprays, eye drops)
- ☐ Household bedroom items (i.e. bedding, mattress protectors, air humidifier)
- ☐ Special clothing (i.e. silk garments, scratch mittens, wet bandages)
- ☐ Kitchen appliances (i.e. bread maker, blender)
- ☐ Over the counter cosmetics (i.e. special suncreams, shampoos, bath products, emollients, moisturisers)
- ☐ Alternative therapies (i.e. Chinese herbal medicine, acupuncture, homeopathic remedies)
- ☐ Other 1 - please specify item below
- ☐ Other 2 - please specify item below
- ☐ Other 3 - please specify item below

Q96

Q96a Other 1 - please specify item [ ]

Q96b Other 2- please specify item [ ]

Q96c Other 3-- please specify item [ ]

On average how much of your own money have you spent on products related to your child's atopic disease (i.e. asthma, food allergies, hayfever or allergic rhinitis)?

		Less than £25	£26-50	£51-100	£101-200	£201-300	£301-£400	£401-500	More than £500
Q97a	Over the counter medications (i.e antihistamines, nasal steroid sprays, eye drops)	○	○	○	○	○	○	○	○
Q97b	Household bedroom items (i.e. bedding, mattress protectors, air humidifier etc)	○	○	○	○	○	○	○	○
Q97c	Special clothing (i.e. silk garments, scratch mittens, wet bandages)	○	○	○	○	○	○	○	○

Q97d	Kitchen appliances (i.e. bread maker, blender etc)	○	○	○	○	○	○	○	○
Q97e	Over the counter cosmetics (i.e. special suncreams, shampoos, bath products)	○	○	○	○	○	○	○	○
Q97f	Alternative therapies (i.e. Chinese herbal medicine, acupuncture, homeopathic remedies)	○	○	○	○	○	○	○	○

		Less than £25	£26-50	£51-100	£101-200	£201-300	£301-£400	£401-500	More than £500
Q97g	{Q96a}	○	○	○	○	○	○	○	○
Q97h	{Q96b}	○	○	○	○	○	○	○	○
Q97i	{Q96c}	○	○	○	○	○	○	○	○

Thank you for taking the time to complete this questionnaire.
