# Supplementary material for: Investigating the health economic burden of atopic disease in children from the EAT‐On Study
Source: Pediatr Allergy Immunol. 2025 Dec 4;36(12):e70256. doi: 10.1111/pai.70256 (PMC12678843; doi:10.1111/pai.70256)
Supplement: Supplementary file 1 — Data S1. [file PAI-36-e70256-s001.zip › pai70256-sup-0002-AppendixS1-TableS3@Supplementary Appendix HEQ.docx]

**Supplementary Appendix**

Supplementary Appendix S1: Health Economics Questionnaire (see attached PDF)

Supplementary Appendix Table S1: Unit costs including any assumptions made.

| Service | Unit cost (UK£2021) | Source and any assumptions made (A) |
| --- | --- | --- |
| Primary care/community services (per contact/visit/appointment) | | |
| GP (per patient contact lasting 9.22 minutes) | 39.23 | *PSSRU: Unit costs of health and social care 2021*  A: Includes qualification costs and carbon emissions |
| GP Nurse (per patient contact lasting 20 minutes – see assumption) | 14.00 | *PSSRU: Unit costs of health and social care 2021*  A: Including qualifications, Unit cost per hour £44.00 divided by 3 to calculate cost per patient visit (i.e. assumed each visit lasts on average 20 minutes) |
| School-based children’s health core (other services) | 80.00 | *PSSRU: Unit costs of health and social care 2021* |
| Physiotherapy | 114.00 | *PSSRU: Unit costs of health and social care 2021* |
| Dietitian | 92.00 | *PSSRU: Unit costs of health and social care 2021* |
| Occupational therapy | 160.00 | *PSSRU: Unit costs of health and social care 2021* |
| Speech therapy services | 114.00 | *PSSRU: Unit costs of health and social care 2021* |
| Other school-based services (i.e. school nurse, other community nurse) | 80.00 | *PSSRU: Unit costs of health and social care 2021* |
| Incontinence service/nurse | 97.65 | *National schedule of NHS costs FY20-21. CHS section.*  A: Average taken of types of community incontinence services |
| Podiatry | 105.82 | *National schedule of NHS costs FY20-21. CHS section.*  A: Average taken of types of podiatry services |
| Community audiology | 157.08 | *National schedule of NHS costs FY20-21. CHS section.*  A: Average taken of types of audiology services |
| Child/adolescent mental health services (per community contact) | 225.00 | *PSSRU: Unit costs of health and social care 2021*  A: One to one session – assumed that this includes psychologists |
| Dental – NHS | 272.21 | *National schedule of NHS costs FY20-21. CHS section.*  A: Average taken of types of community dental services |
| Community paramedic | 89.59 | Inflated from 2016/17 NHS Reference Costs - Currency Code: ASC1. Currency name: Calls. Activity: 1590487. National Average Unit Cost. |
| Health visitor | 85.22 | *PSSRU: Unit costs of health and social care 2021*  A: Average taken of types of health visitor services suitable for a child age 7-12-years old |
| Community optician | 36.69 | NHS reference cost 2011/12 - £31.44  A: Adjusted for inflation using The NHS cost inflation index (NHSCII) from the *PSSRU: Unit costs of health and social care 2021* |
| Community pharmacist | 54.00 | *PSSRU: National schedule of NHS costs FY20-21.*  A: Band 6 Agenda for Change (AFC), cost per working hour. No data regarding length of time spent with HCP so using 1-hour working cost |
| Elective inpatient admissions (paediatrics)   - Average cost per stay | 5244.58 | *PSSRU: Unit costs of health and social care 2021*  A: Average calculated from all available paediatric admissions taking into consideration national average cost and number of data submissions |
| Emergency inpatient admissions (paediatrics)   - Average cost per stay | 3601.76 | *PSSRU: Unit costs of health and social care 2021*  A: Average calculated from all available paediatric admissions taking into consideration national average cost and number of data submissions for non-elective short stay and non-elective long stay (average of the two) |
| Accident and Emergency attendance | 297.00 | *PSSRU: Unit costs of health and social care 2021*  A: Cost for adult and paediatric patients the same |
| Hospital outpatient attendances (average cost per attendance) | | |
| - General Paediatrics | 303.00 | Total Outpatient Attendance section of the *National schedule for NHS costs FY2020-2021 –* for specific paediatric specialty services including clinics and clinical psychology.  A: All clinic encounters were in the outpatient setting and the total unit cost was used |
| - Paediatric clinical immunology and allergy service | 292.00 |  |
| - Paediatric respiratory | 264.00 |  |
| - Paediatric dermatology | 197.00 |  |
| - Paediatric gastroenterology | 239.00 |  |
| - Paediatric Ears, nose, throat (ENT) | 159.00 |  |
| - Paediatric medical oncology | 310.00 |  |
| - Paediatric plastic surgery | 204.00 |  |
| - Clinical genetics | 521.00 |  |
| - Paediatric Ophthalmology | 174.00 |  |
| - Paediatric Trauma & Orthopaedics | 212.00 |  |
| - Paediatric cardiology | 244.00 |  |
| - Paediatric Urology | 147.00 |  |
| - Paediatric Maxillo-facial surgery | 325.00 |  |
| - Paediatric surgeon | 231.40 |  |
| - Paediatric neurology | 337.45 |  |
| - Paediatric nephrology | 345.00 |  |
| - Hospital audiological medicine | 186.00 |  |
| - Radiographer | 59.00 | *Interactive Costing Tool: NIHR Investigations and Intervention Tariff 2020/21 – Section 12: Radiology tariffs*  A: Cost for radiologic examination xray bone imaging (highest tariff used for average) |
| - Hospital EEG | 350.11 | *National schedule for NHS costs FY2020-2021- OPROC section*  A: Conventional EEG for 18 years or under (Paediatrics) |
| - Non-consultant led paediatric services including hospital nurse | 183.69 | Non-consultant led section of the *National schedule for NHS costs FY2020-2021 –* for specific paediatric specialty services including clinics and clinical psychology.  A: Average taken of Paediatric services |
| - Clinical psychology | 222.00 | Total Outpatient Attendance section of the *National schedule for NHS costs FY2020-2021 –* for specific paediatric specialty services including clinics and clinical psychology |
| - Dietitian | 107.00 | Total Outpatient Attendance section of the *National schedule for NHS costs FY2020-2021 –* for specific paediatric specialty services including clinics and clinical psychology |
| - Physiotherapy | 119.00 | Total Outpatient Attendance section of the *National schedule for NHS costs FY2020-2021 –* for specific paediatric specialty services including clinics and clinical psychology |
| - Occupational Therapy | 118.00 | Total Outpatient Attendance section of the *National schedule for NHS costs FY2020-2021 –* for specific paediatric specialty services including clinics and clinical psychology |
| - Speech and language therapy | 166.00 | Total Outpatient Attendance section of the *National schedule for NHS costs FY2020-2021 –* for specific paediatric specialty services including clinics and clinical psychology |
| Prescription costs | | |
| - Cost per item | 8.65 | *Prescription Cost Analysis England 2020-2021*   - Consultation not included within cost of the prescription - Assume only 1 medication per prescription - NHS cost – paediatric prescriptions are free to families - Cost is the same for the prescription in all healthcare settings |
| Auto-injector adrenaline (AAI) device   - Epipen 0.3mg - Jext 0.3mg - Average AAI cost for 0.3mg dose | 67.80  66.30  67.10 | *Prescription Cost Analysis England 2020-2021*  A: Averages taken for costs of each device at each dose   - Assumption that EAT-On children by age will be prescribed 0.3mg device – average taken between Epipen and Jext |
| Gross hourly pay for all employee jobs | 17.79 | *Annual Earnings and Savings Survey, all employees: ASHE Table 1 2021 revised edition of the dataset (Office of National Statistics, UK)*  A: Pay is the same for men and women as parent completing HEQ was not asked to disclose sex |

Supplementary Appendix Table S2: Comparing clinical characteristics between the participants who completed the Health Economics Questionnaire to the participants who did not

|  | Participants who completed HEQ (n=625) | Participants who did not complete HEQ (n=322) | Mean difference (CI) or P-value |
| --- | --- | --- | --- |
| Female n (%) | 326 (52.2) | 154 (47.8) | 0.206 |
| Mean age in years (SD) | 8.5 (1.1) | 9.3 (1.4) | -0.87 (-1.0, -0.7), p<0.001 |
| Ethnicity n (%)   - White Caucasian - Mixed - Asian or Asian British - Black or Black British - Chinese or other ethnic group - White Caucasian - Non-Caucasian | 550 (88.0)  46 (7.4)  9 (1.4)  14 (2.2)  6 (0.96)  550 (88.0)  75 (12.0) | 278 (86.3)  31 (9.6)  5 (1.6)  6 (1.9)  2 (0.6)  278 (86.3)  44 (13.7) | 1.86 p=0.76  -0.02 (-0.06, 0.03) p=0.47 |
| Pet ownership | 257 (41.1) | 147 (45.7) | -0.05 (-0.1. 0.02) |
| Maternal age at leaving full time education n (%)  16 years or younger  17-18 years old  19 years or older  Still studying | 32 (5.1)  72 (11.5)  510 (81.6)  11 (1.8) | 10 (3.1)  48 (14.9)  256 (79.5)  8 (2.5) | -0.1 (-0.08, 0.6)  p=0.704 |
| Paternal age at leaving full time education n (%)  16 years or younger  17-18 years old  19 years or older  Still studying | 81 (13.0)  111 (17.8)  415 (66.4)  18 (2.9) | 44 (13.7)  54 (16.8)  215 (66.8)  9 (2.8) | 0.01 (-0.1, 0.1)  p=0.922 |
| Any history of atopic disease during the EAT study n (%)   - History of atopic dermatitis/eczema - History of wheeze - History of rhinitis | 327 (52.3)  278 (44.5)  109 (17.4)  208 (34.4) | 169 (52.5)  145 (45.0)  55 (17.1)  93 (30.5) | -0.002 (-0.07, 0.07) p=0.96  -0.01 (-0.07, 0.06) p=0.87  0.003 (-0.05, 0.05) p=0.89  0.04 (-0.03, 0.1) p=0.24 |
| Atopic dermatitis SCORAD severity (3 months) n (%)  0 (Absent)  1 (Mild)  2 (Moderate) | n=624*  473 (75.8)  116 (18.6)  35 (5.6) | 239 (74.2)  64 (19.9)  19 (5.9) | -0.02 (-0.1, 0.05)  p=0.64 |
| Atopic dermatitis SCORAD severity (1 year) n (%)  0 (Absent)  1 (Mild)  2 (Moderate) | n=603*  450 (74.6)  125 (20.7)  28 (4.6) | n=306*  217 (70.9)  70 (22.9)  19 (6.2) | -0.05 (-0.1, 0.03)  p=0.19 |
| Atopic dermatitis SCORAD severity (3 years) n (%)  0 (Absent)  1 (Mild)  2 (Moderate) | n=621*  482 (77.6)  103 (16.6)  36 (5.8) | n=314*  256 (81.5)  33 (10.5)  25 (8.0) | 0.02 (-0.06, 0.1)  p=0.67 |
| *The total n is denoted in parenthesis if it differs from the total n of the whole group due to missing data | | | |

Table S3: Clinical characteristics of participants who completed the Health Economics Questionnaire based on their atopic status.

|  | Total number of patients (n=625) | Any atopy (n=215) | No atopy (n=410) |
| --- | --- | --- | --- |
| Female n (%) | 326 (52.2) | 125 (58.1) | 201 (49.0) |
| Mean age in years (SD) | 8.5 (1.1) | 8.4 (1.1) | 8.5 (1.1) |
| Mean number of family members in household (SD) | 2.3 (0.96) | 2.4 (1.0) | 2.3 (0.94) |
| Ethnicity n (%)   - White Caucasian - Mixed - Asian or Asian British - Black or Black British - Chinese or other ethnic group | 550 (88)  46 (7.4)  9 (1.4)  14 (2.2)  6 (0.96) | 172 (80)  22 (10.2)  5 (2.3)  10 (4.7)  6 (2.8) | 378 (92.2)  24 (5.9)  4 (0.98)  4 (0.98)  0 (0) |
| Age mother left full time education n (%)   - 16 years or less - 17-18 years - 19 years or older | 32 (5.1)  72 (11.5)  521 (83.4) | 10 (4.7)  23 (10.7)  182 (84.7) | 22 (5.4)  49 (12.0)  339 (82.7) |
| Age father left full time education n (%)   - 16 years or less - 17-18 years - 19 years or older | 81 (13.0)  111 (17.8)  433 (69.3) | 29 (13.5)  39 (18.1)  147 (68.4) | 52 (12.7)  72 (17.6)  286 (69.8) |
| Any atopic condition at 7-12-years n (%)  - AD  - Allergic rhinitis  - Asthma  - Food allergies | 215 (34.4)  155 (24.8)  84 (13.4)  55 (8.8)  58 (9.3) | 215 (100)  155 (72.1)  84 (60.9)  55 (25.6)  58 (27.0) | 0 (0)  0 (0)  0 (0)  0 (0)  0 (0) |
| Coeliac disease n (%) | 13 (2.1) | 2 (0.9) | 11 (2.7) |
| Timing of completion of HEQ in relation to COVID n (%)  - Pre-COVID  - During/post-COVID | 430 (68.8)  195 (31.2) | 149 (69.3)  66 (30.7) | 281 (68.5)  129 (31.5) |
